# Ability-based Keyboards for Augmentative and Alternative Communication: Understanding How Individuals’ Movement Patterns Translate to More Efficient Keyboards: Methods to Generate Keyboards Tailored to User-specific Motor Abilities

**DOI:** 10.1145/3491101.3519845

**Published:** 2022-04-28

**Authors:** Claire L. Mitchell, Gabriel J. Cler, Susan K. Fager, Paola Contessa, Serge H. Roy, Gianluca De Luca, Joshua C. Kline, Jennifer M. Vojtech

**Affiliations:** Delsys, Inc. and Altec, Inc., Natick, MA, USA; Department of Speech and Hearing Sciences, University of Washington, Seattle, WA, USA; Communication Center of Excellence, Madonna Rehabilitation Hospital, Lincoln, NE, USA; Delsys, Inc. and Altec, Inc., Natick, MA, USA; Delsys, Inc. and Altec, Inc., Natick, MA, USA; Delsys, Inc. and Altec, Inc., Natick, MA, USA; Delsys, Inc. and Altec, Inc., Natick, MA, USA; Delsys, Inc. and Altec, Inc., Natick, MA, USA

**Keywords:** Accessible computing, keyboard design, personalization, augmentative and alternative communication

## Abstract

This study presents the evaluation of ability-based methods extended to keyboard generation for alternative communication in people with dexterity impairments due to motor disabilities. Our approach characterizes user-specific cursor control abilities from a multidirectional point-select task to configure letters on a virtual keyboard based on estimated time, distance, and direction of movement. These methods were evaluated in three individuals with motor disabilities against a generically optimized keyboard and the ubiquitous QWERTY keyboard. We highlight key observations relating to the heterogeneity of the manifestation of motor disabilities, perceived importance of communication technology, and quantitative improvements in communication performance when characterizing an individual’s movement abilities to design personalized AAC interfaces.

## 1 INTRODUCTION

An estimated 5 million Americans have speech impairments that require the use of augmentative and alternative communication (AAC) technology to meet their daily communication needs. Many AAC users rely on computers, tablets, or smartphones to supplement or replace their oral speech [1, 6, 12]. However, some individuals with concomitant motor disabilities—such as those with cerebral palsy, amyotrophic lateral sclerosis, Parkinson’s disease, and traumatic brain injury, among others—lack the manual dexterity necessary to control mainstream AAC technology. Instead of multi-input methods (e.g., ten-finger typing on a keyboard), these individuals must rely on alternative, single-input modalities such as eye-tracking, head-tracking, and switch-scanning to effectively access virtual interfaces.

Unfortunately, current alternative communication technologies that incorporate single-input access and keyboard interfaces offer limited versatility and personalization for those with motor disabilities that result in severe dexterity impairments. For this population, text is primarily generated using a virtual keyboard. Most interfaces utilize the standard QWERTY layout, which has been described as “grossly inefficient” for single-input use for AAC [26]. Moreover, these devices often require extensive setup and maintenance by a caregiver, frequent recalibrations, and manual interface customizations that burden both caregiver and AAC user, leaving many individuals poorly served. Users may attempt to compensate using their own residual motor capabilities or through substantial reliance on caregiver support and troubleshooting [7]. These and other factors contribute to the nearly one-third of people who abandon their prescribed AAC device in favor of less effective dysarthric speech, gestures, among other communication methods [2, 13].

The communication potential of AAC interfaces has improved in recent years with the inclusion of basic automation features such as word prediction, abbreviation expansion, and the ability to save frequently used words and phrases. However, highly ubiquitous interface software such as Communicator 5 (Tobii Dynavox; Pittsburgh, PA, USA), Proloquo4Text (AssistiveWare; Amsterdam, the Netherlands), or Verbally (Intuary; San Francisco, CA, USA) still require time-consuming, manual support from a trained caregiver to accommodate common changes to personalize a keyboard to meet the needs of the user. The computational optimization of a computer interface to fit the abilities of the user can improve performance [9, 10], yet no existing AAC technology can *automatically* tailor a single-input keyboard interface to an individual’s unique motor abilities. Existing research has examined the effects of configuring a keyboard to given population (e.g., individuals with tremor) [24], yet this approach ignores any heterogeneity in motor abilities *within* the population [14, 22]. Prior work has also examined the use of automated models for classifying keypresses on virtual keyboards [8]; however, these models were constructed for multi-input typing on a QWERTY keyboard. Thus, in this work, we build on the principle of ability-based design to develop and evaluate an AAC system that characterizes an individual’s cursor control abilities to automatically personalize the layout of a keyboard interface for improved communication. We present three use cases involving people with diverse motor disabilities who use this AAC system to create messages, comparing performance to the ubiquitous QWERTY keyboard and a computationally optimized but non-personalized keyboard.

## 2 ABILITY-BASED KEYBOARD GENERATION AND EVALUATION

This work expands on novel methods for ability-based keyboard generation in individuals without dexterity impairments [23], as well as prior work on generically optimizing a keyboard to improve communication in individuals with dexterity impairments due to motor disabilities [4]. Here, we examine the feasibility of ability-based keyboard generation for those with motor disabilities. We briefly summarize (see [23]) steps to algorithmically characterize movement and generate personalized keyboards below.

### 2.1 Characterizing User Cursor Control

Our methods for characterizing cursor control abilities expand on Fitts’ Law [16, 17] to estimate movement time and distance relationships relative to a given target angle rather than the typical approach of grouping time and distance data irrespective of angle. Users are presented with a modified version of the multidirectional Fitts’ Law-based point-select task, wherein a grid of hexagonal keys are configured on a computer screen and users must navigate to and select a highlighted key (or “target”). Targets are seeded to capture two-dimensional (2D) movements across a range of possible distances and directions. Each target is categorized into one of 16 pre-specified bins spanning (360/16 =) 22.5 degrees by the empirical angle of target selection relative to the previous target [23]. Within each bin, a linear regression is performed to yield constants a and b from Mackenzie’s Shannon formulation of Fitts’ Law (Equation 1) [16, 17]:

(1) $$MT=a+b*ID,\;\text{where ID}={\text{log}}_{2}\left(\frac{D}{W}+1\right)$$

Specifically, the movement time (MT; sec) to select a given target is calculated as the travel time of the cursor between those clicks; distance (D; pixels) is calculated as the Euclidean distance between those click locations. Each distance D is converted to an index of difficulty (ID; bits) with W representing a constant target width (pixels).

### 2.2 Generating a Personalized Keyboard Interface

Personalized keyboards are created by leveraging character-to-character (digraph) transition occurrences of a selected corpus, MT (calculated as in Equation 1 with respect to target width and movement distance), and target selection angle. The user-specific a and b constants derived from movement data are sampled relative to the angle of target selection, then applied to Fitts’ Law (Equation 1) to estimate MT and solve the quadratic assignment problem (QAP) via the Fast Approximate QAP Algorithm [27] of the GraphMatch function in graspologic (Microsoft, Redmond, WA, USA) [3]. Thus, each keyboard computationally prioritizes common letter transitions to be in directions easier for the user.

## 3 SYSTEM EVALUATION

### 3.1 Overview

Text input performance was evaluated for each participant when using a keyboard generated via our personalization methods versus that when using QWERTY or a generic, computationally optimized keyboard. To enable comparisons with QWERTY, the width of the space key on the QWERTY keyboard was set equal to all other keys and positioned to the right of the “M” key (see [23]), as prior work has shown that text input performance does not significantly differ in this configuration [23]. Distinct from the personalized keyboards, which are derived from user-specific a and b constants that vary according to movement direction, the generically optimized keyboard was generated using standard directionally static Fitts’ constants of a = 0.127 sec and b = 1/4.9 sec/bits across all target selection angles [21, 30]. Digraph transition occurrences for both the personalized and generically optimized keyboards were calculated from a standard corpus for evaluating text-entry techniques [20]. Computer access was standardized across participants via a validated access method [11] comprising an inertial measurement unit (IMU) for cursor movement (activated via head tilts) and surface electromyographic (sEMG) sensor for cursor clicks (activated via winks or blinks).

### 3.2 Experimental Protocol

Three participants with motor impairments (1 female, 2 male; 24–65 years) completed 1–2 sessions based on availability. All individuals gave informed consent in compliance with the Madonna Rehabilitation Hospital Institutional Review Board (P1 and P2) or Western Institutional Review Board (P3) through either written consent or verbal consent witnessed by a communication partner as appropriate. Of the diagnoses (see Table 1), two were congenital (cerebral palsy; P1, P2) and one was acquired (Parkinson’s disease; P3). All participants reported that they primarily rely on their dysarthric speech for face-to-face communication. P1 also reported using eye-tracking (e.g., for writing school assignments or e-mails) and occasionally using their nose for touchscreen-based navigation.

For each participant, the inertial sensing component of the hybrid sEMG/IMU access method was secured to the center of the forehead—with the y-axis of the IMU parallel to the transverse axes of the head—and the EMG sensing component applied over the orbicularis oculi of the preferred eye. Computer access thresholds were calibrated by instructing each participant to comfortably tilt their head to the left and right twice, up and down twice, and wink or hard blink twice [11, 28]. These data were used to tune the 2D range of cursor movement, as described in detail in [28].

Participants then carried out a movement characterization task within a 9×9 honeycomb grid on a computer monitor with a 1920×1080 resolution to capture movement trajectories across a range of movement distance and directions when using the sEMG/IMU access method. Within the task, participants were instructed to navigate to and select the highlighted target as quickly and as accurately as possible [31]. Participants completed this movement characterization task in 5-minute intervals until 30 minutes had been reached. Participants were allowed ample break time between each interval to minimize fatigue. The task terminated early (i.e., before the 30-minute threshold) if each angle bin contained 5 or more targets and exhibited at least a weak MT-ID correlation (R2 > 0.09 [5]). After completing the task, the 16 angle-specific Fitts’-based a and b constants were used to generate a personalized keyboard.

Each participant was then presented with the generically optimized keyboard and their personalized keyboard in a pseudorandomized order (i.e., generically optimized first or personalized first). Based on their assigned order, participants spelled out a subset of balanced phrases from the corpus used for digraph occurrence calculations [20] in alternating blocks. A final block using the QWERTY keyboard was performed to serve as a reference for communication performance (i.e., due to widespread familiarity in using the QWERTY keyboard for communication) once participants were familiar with the access method. Blocks comprised 10 minutes of interaction with one interface—not including participant-initiated breaks between sentences—followed by a survey to capture their experiences on a 10-cm visual analog scale [4]. The survey included questions about how fast it felt (where 0 cm is anchored as “Very slow” and 10 cm as “Very fast”) to use the generically optimized, personalized, and QWERTY keyboards, as well as how *easily* they understand the keyboard layout (0 cm as “Not easily” and 10 cm as “Very easily”) as an estimate of keyboard familiarity. After each survey, participants could take a break (10–20 minutes, as necessary) to minimize fatigue. At the end of the study, participants were surveyed on which of the two new interfaces they preferred (0 cm as “First Interface” and 10 cm as “Second Interface”).

### 3.3 Data Analysis

User control was examined in the movement characterization task via a series of target-to-target path trajectory metrics. Metrics included an estimate of target-to-target movement velocity, as well as standard, path-specific metrics of movement error, path efficiency, target re-entry, and selections per target [19, 25]. Keyboard communication task data were analyzed to assess communication performance via metrics of speed (words per minute, or WPM), target selection accuracy (%), information transfer rate (ITR; bits per minute) [29], and throughput (bits/min) [18]. Survey responses were also tabulated to assess user effort and preferences and are reported from 0 to 10 cm.

## 4 MOVEMENT CHARACTERIZATION

### 4.1 Target-to-target Path Trajectories

**Figure 1:**
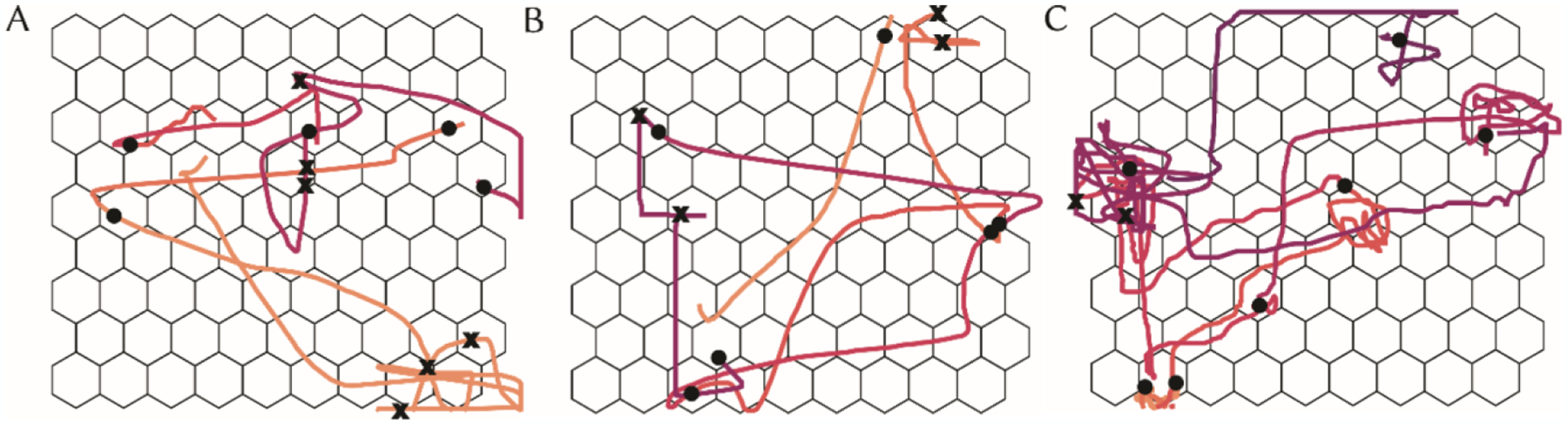
Example path trajectories for accurate (black circle) and inaccurate (black ‘x’) clicks for A) P1, B) P2, and C) P3.

Participant target-to-target path trajectories resulting from our movement characterization task successfully show variations in 2D cursor control. These differences are exemplified in the path trajectories shown for each participant in Figure 1 and are further summarized via path trajectory metrics in Table 2.

Each participant traveled at substantially different movement velocities when traveling between targets, wherein P3 navigated the fastest, followed by P2 then P1. P3’s fast cursor movements are further characterized by multiple target overshoots, which accounts for the observed low mean path efficiency. P2’s target-to-target movements were highly efficient, indicating precise movement with low target re-entries, yet large deviations from the target axis through a high movement error, suggesting a preference toward independent movements in *x* and *y* directions. Despite P2’s propensity to not overshoot the target, they often required more than one selection to accurately click the target. In addition to slow cursor movements, P1’s path trajectory results also demonstrate a slight preference toward coordinated 2D movements with minimal deviation from the task axis (via movement error) and a moderate mean path efficiency. On average, P1 exhibited approximately one target overshoot and more than one selection to accurately click the target.

Participants required an average of five 5-minute intervals to complete the movement characterization task (P1: 4, P2: 5, P3: 6). Notably, all participants exhibited large movement errors (138.0–172.9 pixels) compared to those in the literature for people with motor disabilities when using computer pointing devices (30.4 pixels [15]); this is likely due to differences in access method (sEMG/IMU vs. computer mouse) and experimental methodology (e.g., task instructions).

### 4.2 Relationship between Movement Time and Direction

**Figure 2:**
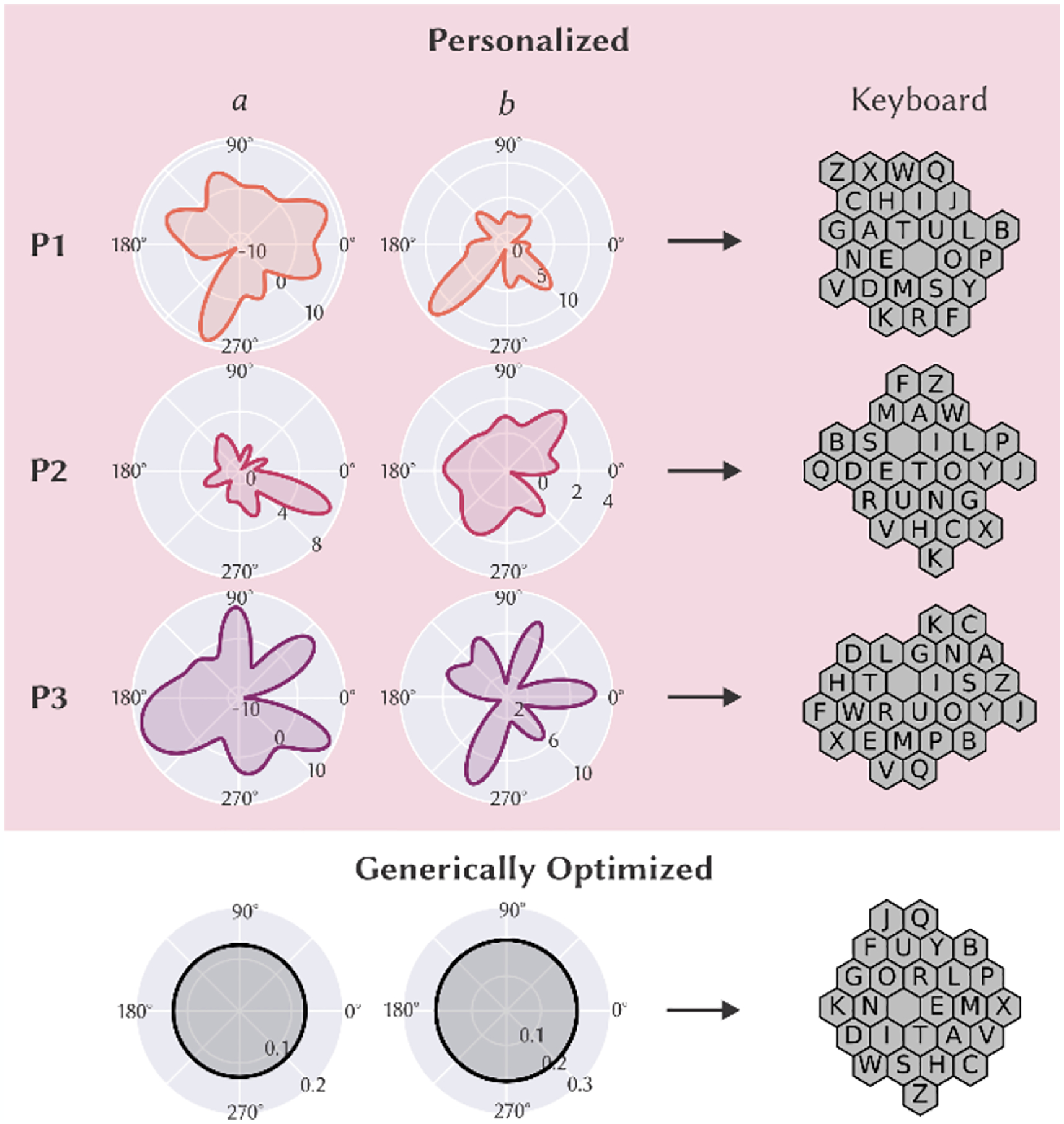
Fitts’ constants a (left; sec) and b (middle; sec/bit) and resulting keyboards (right) for each participant (rows) relative to the generically optimized keyboard (bottom row).

The movement characterization algorithms effectively captured differences in movement time relative to distance and direction. On average, P3 required 10.2 sec (*SD* = 7.9 sec) to travel between targets, whereas P1 required 7.4 sec (*SD* = 5.9 sec) and P2 required 5.3 sec (*SD* = 3.5 sec). MT-ID relationships were moderate-to-strong for each participant when accounting for movement direction (*R*^*2*^ = 0.35 for P1, *R*^*2*^ = 0.44 for P2, and *R*^*2*^ = 0.25 for P3) [5]; resulting values for Fitts’ constants *a* and *b* are shown for each participant relative to movement direction (0 to 360 degrees) in Figure 2.

Using these derived sets of *a* and *b* constants, a personalized keyboard was then generated for each participant (see section 2.2) to reflect their movement abilities. The keyboard for P1 reflects the larger movement times required to navigate diagonally down and to the left via organizing fewer keys in that region of the keyboard. The keyboard for P2 is arranged in a cross-like pattern, which reflects P2’s large movement errors and preference for moving in independent (1D) *x*/*y* directions (i.e., by allowing for more independent *x*/*y* movements than would a compact, circular keyboard), and is also demonstrated via reduced *a* and *b* values in each cardinal direction in Figure 2. The keyboard for P3 reflects reduced *a* and *b* constants within the horizontal plane, as the keyboard is wider than generically optimized keyboard; this configuration describes a preference for P3 to move left and right (i.e., rather than up or down). Contrastingly, the generically optimized keyboard shows effect of directionally static constants its circular geometry.

## 5 KEYBOARD COMMUNICATION

**Figure 3:**
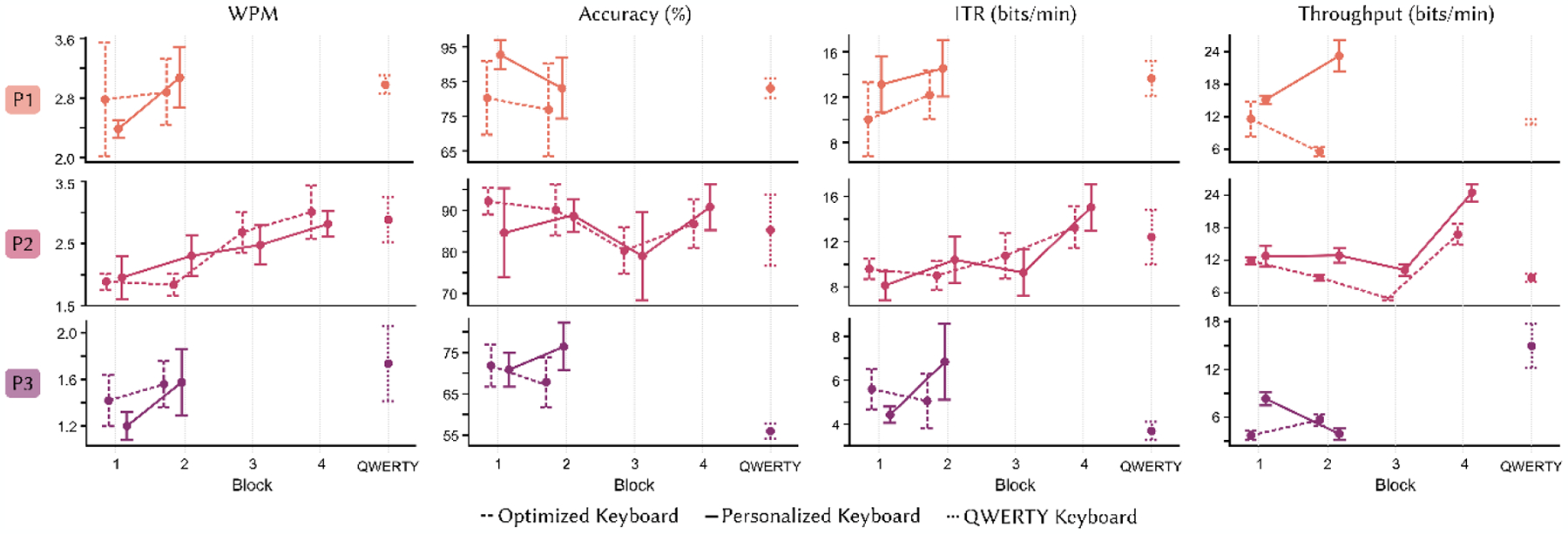
Communication performance via WPM (left), accuracy (middle), and ITR (right) across block for participants (rows) when using generically-optimized (dashed line), personalized (solid line), and QWERTY (dotted line) keyboards.

Keyboard communication results for personalized, optimized, and QWERTY keyboards are shown in Figure 3.

P1 completed two blocks each with generically optimized (presented first) and personalized (presented second) keyboards, followed by one block with the QWERTY keyboard. P1 demonstrated superior communication performance when using their personalized keyboard compared to either the optimized or QWERTY keyboards for WPM, ITR, and throughput. When surveyed about each keyboard, P1 consistently reported that it felt faster to use their personalized keyboard (7.4±1.0 of 10 cm) than either the optimized (4.3±0.2 of 10 cm) or QWERTY (6.6 of 10 cm) keyboards. P1 additionally indicated the most familiarity with QWERTY (10 of 10 cm), but that it was easier to understand the layout of their personalized keyboard (7.9±1.1 of 10 cm) than the optimized keyboard (6.2±0.7 of 10 cm). P1 ultimately designated a strong preference for their personalized keyboard over the generically optimized keyboard (9.5 of 10 cm in favor of personalized).

P2 was presented with their personalized keyboard first followed by the generically optimized keyboard, completing blocks 1 and 2 in one day and blocks 3 and 4 in a second day alongside the QWERTY block. P2 showed superior performance when using their personalized keyboard during the final block for each day (i.e., blocks 2 and 4); this is demonstrated via higher WPM, throughput, and ITR on the first day and by higher accuracy, throughput, and ITR on the second day. Communication performance during the final exposure to the personalized keyboard (block 4) exceeded that of the QWERTY board through accuracy, throughput, and ITR. Subsequently, P2 reported that it felt slightly faster to use the personalized (4.8±0.2 of 10 cm) and optimized (4.8±0.2 of 10 cm) keyboards compared to the QWERTY keyboard (4.7 of 10 cm); however, the speed-of-use ratings were quantitatively similar across all three keyboards. P2 also reported that it was easiest to understand the layout of QWERTY (9.7 of 10 cm), followed by their personalized keyboard (6.2±1.0 of 10 cm), then the optimized keyboard (5.9±1.1 of 10 cm). Following this, P2 strongly preferred their personalized keyboard over the generically optimized keyboard (10 of 10 cm in favor of personalized).

P3 completed two blocks each for personalized (presented first) and generically optimized (presented second) keyboards, followed by one block with QWERTY. In their final exposure to the new keyboards, P3 demonstrated better performance using their personalized board in terms of accuracy and ITR. P3’s final communication performance when using their personalized keyboard exceeded that of QWERTY through accuracy and ITR. Survey results were only collected after the second block for personalized and optimized keyboards. Dissimilar from their text input performance, P3 reported that it felt slightly faster to create messages using the optimized keyboard (3.5 of 10 cm) compared to their personalized keyboard (3.2 of 10 cm), and that both new keyboards felt faster to use than QWERTY (2.2 of 10 cm). P3 additionally reported that it was as easy to understand the layout of their personalized keyboard as of QWERTY (9.0 of 10 cm). P3 found it most difficult to understand the optimized keyboard layout (7.7 of 10 cm) and, accordingly, exhibited a strong preference for their personalized keyboard (10 of 10 cm in favor of personalized).

## 6 SIGNIFICANCE & FUTURE DIRECTIONS

The results presented here provide insight into the communication benefits that may be achieved through automatic, ability-based generation of keyboards for individuals with motor disabilities. By examining cursor movements during a 2D multidirectional point-select task, this work has yielded an automated method to characterize user movement patterns and abilities that may be used to control a computer cursor. Unique geometric organization of the personalized keyboards visibly reflected the diverse movement patterns observed in the movement characterization, highlighting the immense heterogeneity of the manifestation of motor disabilities. The personalized keyboards also showed benefits across participants via ITR—a metric that unifies both speed and accuracy—but not throughput. These findings indicate clear communication benefits in some users while also emphasizing the importance of using multiple metrics to comprehensively examine text input performance. Finally, this work highlights the importance of user perception in AAC: even with variability in performance across keyboards, all three participants reported a strong preference for their personalized keyboard and, furthermore, that using their new, personalized keyboard felt faster than QWERTY.

Given these promising results, we intend to expand testing among a larger, diverse group of prospective users who require alternative communication technology due to developmental disabilities, acquired neurogenic disorders, and/or degenerative neurological conditions to further elucidate the utility of our methods. We also aim to conduct a field study to examine the ecological validity of our keyboard generation algorithms when used to create a personalized keyboard based on a user’s preferred access method (e.g., eye- or head-tracking device, joystick) instead of the sEMG/IMU method examined here, which was new to participants. Finally, it is important to note that although ITR was higher using the personalized keyboard over QWERTY, overall text input performance was of similar magnitude between the new keyboards (optimized, personalized) and QWERTY. This, especially emphasized through speed (WPM), is likely the result of overtrained experience with QWERTY and undertrained experience with the new keyboards (each used for ~1 hour). These results are in line with prior work suggesting overtraining effects will likely be overcome after 4–5 hours of interaction [4], indicating potential increases in text input performance using the personalized keyboards. Future work should examine the longitudinal effects of training with the new keyboards compared to QWERTY.

## 7 CONCLUSION

Current AAC interfaces require tedious, manual support from a trained caregiver to personalize a keyboard to meet user needs. In this work, we extended the use of ability-based methods to individuals with heterogenous motor disabilities to determine the utility of automatically personalizing a keyboard to user-specific motor abilities. In doing so, we observed diverse movement capabilities as individuals navigated through different geometric organizations and shapes to create messages on virtual keyboards. Communication performance improved in all participants when using their personalized keyboard compared to QWERTY or a generically optimized keyboard. These results highlight the feasibility of automated, ability-based techniques as a valuable tool for generating individualized keyboards that accommodate the diverse population of individuals who must rely on alternative technologies for communication.

## Figures and Tables

**Table 1:** Demographics of participants with motor impairments, including primary communication modalities.

ID	Age/sex	Diagnosis	Communication Method(s)	Characteristics
P1	24/F	Cerebral Palsy	Oral speech, eye tracking, or nose on touchscreen	Involuntary spasms of the lower facial musculature
P2	40/M	Cerebral Palsy	Oral speech, fingers on touchscreen	Neck rigidity, limited ability to tilt head left
P3	65/M	Parkinson’s Disease (16 yrs post-diagnosis)	Oral speech	Prominent bilateral resting and action tremor, difficulty coordinating head movement and eyeblinks

**Table 2:** Target-to-target Path Trajectory Results

Metric^a^	Participant		
	P1	P2	P3
Movement Velocity (pixels/sec)	42.8 ± 22.5	64.6 ±23.9	134.8 ±40.7
Movement Error (pixels)	138.0 ± 114.1	172.9 ± 131.4	149.6 ± 112.6
Path Efficiency (%)	57.8 ± 26.4	72.9 ± 21.0	39.5 ± 23.9
Target Re-entry	1.1 ± 1.9	0.3 ± 1.0	2.5 ± 3.5
Selections per Target	1.3 ± 0.6	1.6 ± 1.2	1.2 ± 0.7

a Movement characterization data are reported as mean ± standard deviation.

## CCS CONCEPTS

- **Human-centered computing** → Accessibility; Accessibility design and evaluation methods; Interaction design; Interaction design process and methods; User interface design; Human computer interaction (HCI); Interaction devices; Keyboards.
